# The 3-year follow-up of a fully biodegradable implantable device closure for perimembranous ventricular septal defects in children using echocardiography

**DOI:** 10.3389/fcvm.2024.1420704

**Published:** 2024-07-02

**Authors:** Juan Cong, Cunying Cui, Danqing Huang, Ying Wang, Sifan Liu, Shubo Song, Taibing Fan

**Affiliations:** ^1^ Department of Ultrasound, Fuwai Central China Cardiovascular Hospital, Fuwai Central China Hospital of Zhengzhou University, Henan Provincial People’s Hospital Heart Center, Zhengzhou, Henan, China; ^2^ Department of Children’s Heart Center, Fuwai Central China Cardiovascular Hospital, Fuwai Central China Hospital of Zhengzhou University, Henan Provincial People’s Hospital Heart Center, Zhengzhou, Henan, China

**Keywords:** fully biodegradable occluder, ventricular septal defect, follow-up, echocardiography, myocardial deformation

## Abstract

**Objects:**

The aim of this study was to investigate the morphologic changes of a novel fully biodegradable implantable device after closing a perimembranous ventricular septal defect (Pm-VSD) and to evaluate the effect of the occluder on the myocardial function in patients during a 3-year follow-up period.

**Methods:**

One-year, 2-year, and 3-year follow-ups were carried out after implantation with a total of 30 Pm-VSD patients who had successful closure by the fully biodegradable occluder. In total, 30 healthy children were enrolled as controls. At discharge and at every follow-up visit, the lengths of the left and right discs of the novel device were measured in the apical three- and four-chamber as well as short-axis views. At the end of the follow-up, using three-dimensional speckle-tracking conditions, the values of myocardial deformation, including global longitudinal strain, global circumferential strain, and global area strain, were acquired.

**Results:**

The fully bioabsorbable double-disc occluder gradually decreased over time and was eventually invisible under echocardiographic scanning during the follow-up (*p *< 0.05). At the end of the third year, there were no significant differences in the myocardial deformation parameters between the cases implanted with the novel devices and the controls; no significant differences were found between the basal segments of the ventricle septa and that of the left ventricle (LV) free wall among the patients who completed the Pm-VSD closure using the fully biodegradable occluder (*p *> 0.05).

**Conclusion:**

The novel fully biodegradable occluder is a safe, effective, and perfect alternative for the treatment of VSD. Echocardiography plays a crucial role in the follow-up of this new type of occluder implantation.

## 1 Introduction

Ventricular septal defect (VSD) is one of the most common congenital heart diseases (1). In recent decades, with the improvement of interventional technology and instruments, especially since the AMPLATZER Septal Occluder was applied in clinic in 1998, transcatheter therapy has become an attractive choice of treatment for common congenital heart diseases, such as VSD, atrial septal defect, and patent ductus arteriosus (2–4).

However, clinical follow-up has found that because of the composition of the Ni-TI alloy, with a nickel content of approximately 55%, metal occluders can lead to late-onset adverse reactions, such as allergies. In addition, owing to the long-term insertion in the heart and constant pressing on the surrounding tissues, the traditional device may cause chronic inflammation, cardiac tissue abrasion, valve perforation, and other serious complications, such as high atrioventricular block (5–8).

For this reason, bioresorbable medical devices have been rapidly developed in recent years. A fully biodegradable polydioxanone occluder for VSD closure with good short- and mid-term performance was previously reported (9, 10). In this study, using echocardiography, we performed a cohort study to investigate the changes in morphology of a novel fully biodegradable implantable device after closing perimembranous VSD (Pm-VSD) in 30 children during a 3-year follow-up, and to evaluate the effect of the occluder on local myocardial function in patients at the end of the third year visit.

## 2 Materials and methods

### 2.1 Study subjects

This was a prospective cohort study approved by the ethics committee of the hospital (2019-Q009-01). All parents of the children provided signed informed consent. Between October 2019 and May 2023, a total of 34 children with Pm-VSD diagnosed using transthoracic echocardiography underwent implantation of the fully biodegradable occluder. The inclusion criteria were isolated Pm-VSD with the right-sided opening diameter ≥3 and ≤14 mm; the upper margin of VSD ≥3 mm from the aortic valve; and, in principle, age ≥1 year and weight ≥10 kg. The exclusion criteria were aortic valve prolapse, multiple VSD, no obvious edge of the defect, combined with other congenital diseases, infective endocarditis, and severe pulmonary hypertension.

Of the 34 individuals initially registered for the study, four had failed operations and were excluded. Finally, 30 patients with successful Pm-VSD closure were enrolled in the study. For the cases in this cohort, the global follow-up period was 3 years. Our outcome variables were 1-year, 2-year, and 3-year follow-ups after implantation. At every visit, all patients underwent laboratory examinations (including routinely hematologic parameters and biochemical blood indices), transthoracic echocardiogram, and electrocardiograms. To avoid radiation exposure, chest radiography was not performed unless necessary. In addition, 30 healthy children matched for sex, age, and body surface area were included as controls to compare myocardial function between the cases and controls at the end of the third year.

### 2.2 Occluder device and delivery system and treatment

**Figure 1:**
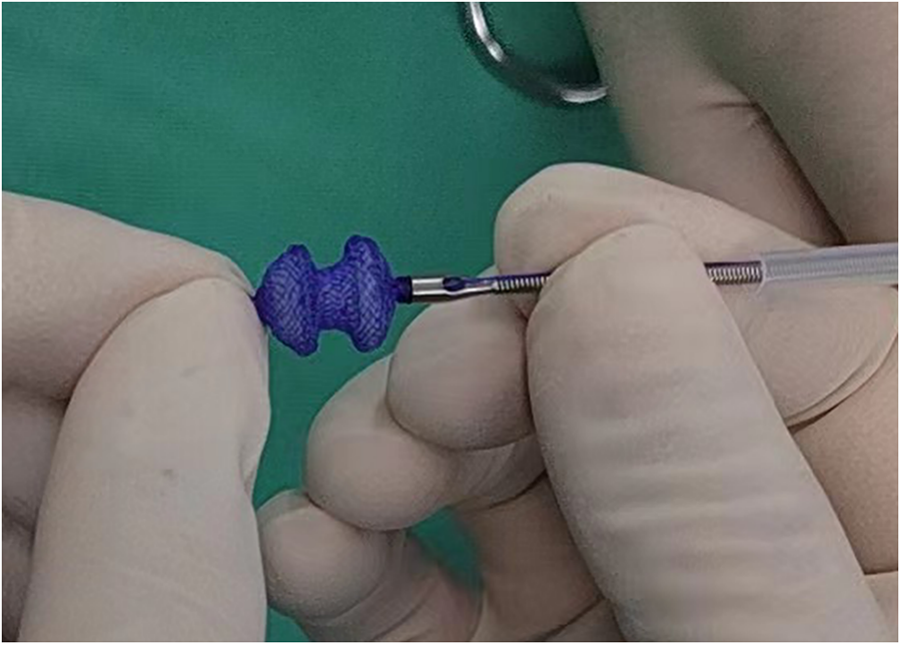
The fully biodegradable occluder and delivery system (Shanghai Shape Memory Alloy Co. Ltd.). The occluder was a double-disc waist drum structure with a symmetrical wide edge. The skeleton frame was braided by a polydioxanone monofilament and the occlude membrane was composed of poly-L-lactic acid.

The fully biodegradable occluder and delivery system (Shanghai Shape Memory Alloy Co. Ltd., Shanghai, China) used in this study was developed and reported initially by Chen et al. (9, 11). The occluder was a double-disc waist drum structure with a symmetrical wide edge and single riveted concave disc surface (Figure 1). The disc diameter was approximately 2–3 mm larger than the waist and the lumbar diameter matched VSD size. The skeleton frame was braided by a polydioxanone monofilament and the occlude membrane was composed of poly-L-lactic acid.

Implantation was achieved via the minimal right subaxillary incision-right atrial thoracotomy. The detailed procedure of the operation has been described previously (10). Since the novel occluder was free from x-ray radiation, the whole operation was carried out under the guidance and monitoring of transesophageal echocardiography (TEE) only. All patients received oral aspirin (5 mg/kg/day) postoperatively for 6 months.

### 2.3 Echocardiography

An ultrasound was performed using a system equipped with transesophageal transducers of 8–3 and 6–10 MHz (Vivid E95; GE Vingmed Ultrasound AS, Horten, Norway), depending on patient size, before, during, and after the implanting procedure with the fully biodegradable occluder for closure of the Pm-VSD.

At discharge and at every follow-up visit, according to the current recommendation (12), the participants underwent extensive transthoracic echocardiography using a 4VC transducer (1.4–5.2 MHz; GE Vingmed Ultrasound, Horten, Norway). All image recordings consisting of three consecutive cardiac cycles with a frame rate of 60–80 s^−1^ were obtained and saved in cine-loop digital format for offline analysis (EchoPAC; GE Healthcare, Horten, Norway).

The diameters of interventricular septum, posterior wall, and left ventricle (LV) diameters at end-diastole and end-systole via M-Mode were measured in the parasternal long-axis view, then LV ejection fraction and stroke volume were calculated. The lengths of left and right discs of the novel device were measured in the apical three- and four-chamber as well as short-axis views, respectively. At the end of the 3-year follow-up, using three-dimensional speckle-tracking conditions, the values of regional and global strain were presented as strain curves and a color-coded 17-segment bull's eye plot. Then, global longitudinal strain (GLS), global circumferential strain (GCS), and global area strain (GAS) were calculated as previously reported (13).

### 2.4 Statistical analysis

The statistical analyses were performed using SPSS version 26 (SPSS Inc., Armonk, NY, USA). All data were expressed as mean ± standard deviation for continuous variables, and frequencies or percentages for nominal variables appropriately. Myocardial deformation data were presented as their absolute values. Differences between the follow-up groups were analyzed for statistical significance using paired *t*-tests with Bonferroni correction, while independent sample *t*-tests were utilized to assess the differences in myocardial function between the Pm-VSD cases with successful closure and the controls at the end of the 3-year visit. A *p*-value < 0.05 was considered statistically significant.

## 3 Results

### 3.1 Clinical and echocardiographic characteristics of the Pm-VSD children with successful closure

Among 34 cases suffering from Pm-VSD, 30 patients (15 boys, 15 girls) were treated successfully. Their mean age was 3.41 ± 2.29 years (range 3 months–10 years), mean height was 97.38 ± 16.99 cm (range 73–131 cm), and mean weight was 15.08 ± 5.22 kg (range 8–25.6 kg) (Table 1). The size of occluded VSDs was in the range of 3.0–6.0 mm, with a mean of 4.70 ± 1.05 mm, while the mean VSD diameter measured using echocardiography was 4.97 ± 1.67 mm. The minimum distances from the aortic valve and from the tricuspid septum were 2.80 and 1.50 mm, respectively. Meanwhile, the mean waist size of the double-disc device was 5.80 ± 1.30 mm (range 4.0–8.0 mm). With a “double-umbrella” configuration, the occluders clamped and closed the defects while it located precisely and fixed well postoperatively. Moreover, the device did not impede the opening and closing of the aortic and tricuspid valves.

**Table 1:** Clinical and echocardiographic data of the Pm-VSD cases with successful closure.

Variables	Data
Sample size (number)	30
Male (number)	17
Age (years)	3.41 ± 2.29 (1.6–10.0)
Height (cm)	97.38 ± 16.99 (70–131)
Weight (kg)	15.35 ± 5.05 (8.5–25.6)
Pm-VSD size measured by TEE (mm)	4.97 ± 1.67 (2.7–8.9)
Distance from aortic valve by TEE (mm)	4.42 ± 2.21 (2.8–12.0)
Distance from tricuspid septum by TEE (mm)	3.89 ± 2.00 (1.5–8.1)
Pm-VSD size in the operation (mm)	4.70 ± 1.05 (3.0–6.0)

Continuous data are presented as the mean ± standard deviation (range).

Because of failed operations, four individuals were excluded from the study. Among them, two cases terminated the procedure due to a mismatch between the small defects (≤3 mm) and the large sheath tube. They were treated with a Ni-TI occluder device and underwent surgical repair under extracorporeal circulation, respectively. Meanwhile, one case was characterized by multiple defects of aneurismal Pm-VSD and another patient with an unexpectedly large defect (measured at 8.4 mm preoperatively but 12 mm intraoperatively) underwent ventricular septal repair with cardiopulmonary bypass.

No serious complications occurred in those children. At every point, the quantitation of blood laboratory tests, such as white blood cells (WBCs), red blood cells (RBCs), hemoglobin, alanine transaminase (ALT), aspartate transaminase (AST), alkaline phosphatase as well as creatinine, was in normal ranges (Table 2). There were no instances of left and right bundle branch block (RBBB) or atrioventricular block except for one case with incomplete RBBB at discharge but had recovered at her 1-year follow-up. During the follow-up period, a transthoracic echocardiogram was performed and the general measurements were recorded (Table 3). There were no occurrences of residual shunt, device dislocation, new or aggravated valve regurgitation, thrombosis, or infective endocarditis.

**Table 2:** Data of blood laboratory tests at every visit point during follow-up.

Variables	Normal range	At discharge	1-year follow-up	2-year follow-up	3-year follow-up
		(*n* = 30)	(*n* = 30)	(*n* = 24)	(*n* = 13)
WBC (10^9^/L)	5.10–13.20	8.12 ± 1.58	8.19 ± 1.62	8.07 ± 1.40	8.15 ± 1.49
RBC (10^12^/L)	4.00–5.20	4.10 ± 0.50	4.33 ± 0.58	4.29 ± 0.55	4.64 ± 0.50
Hemoglobin (g/L)	107–138	108.00 ± 11.42	114.13 ± 16.16	113.68 ± 14.24	123.00 ± 16.24
Platelets (10^9^/L)	180–486	322.92 ± 109.21	301.21 ± 95.60	314.26 ± 103.89	282.59 ± 80.36
ALT (U/L)	7–40	14.00 ± 3.89	16.32 ± 12.42	14.27 ± 4.04	17.57 ± 14.56
AST (U/L)	13–35	28.92 ± 7.41	29.83 ± 9.26	28.58 ± 6.49	29.82 ± 9.42
Total bilirubin (μmol/L)	0–21	4.35 ± 1.66	5.07 ± 2.02	5.31 ± 2.59	6.43 ± 2.55
Urea (mmol/L)	2.76–8.07	4.00 ± 1.05	3.98 ± 1.05	4.12 ± 1.01	4.09 ± 1.11
Creatinine (μmol/L)	14–34	27.54 ± 6.19	27.42 ± 5.73	27.95 ± 5.89	27.82 ± 5.39

Continuous data are presented as the mean ± standard deviation.

**Table 3:** Routine echocardiographic measurement at every follow-up.

Variables	At discharge	1-year follow-up	2-year follow-up	3-year follow-up
	(*n* = 30)	(*n* = 30)	(*n* = 24)	(*n* = 13)
LVDd (mm)	29.15 ± 3.31	30.38 ± 3.64	31.92 ± 3.80	34.33 ± 5.21
LVDs (mm)	19.08 ± 2.66	19.23 ± 2.65	21.42 ± 1.98	21.82 ± 3.73
IVS (mm)	4.93 ± 0.45	4.62 ± 0.64	5.11 ± 0.98	5.33 ± 0.90
LVPW (mm)	4.91 ± 0.46	4.58 ± 0.60	5.09 ± 0.86	5.30 ± 0.72
LA (mm)	19.23 ± 2.01	19.58 ± 2.43	19.62 ± 2.18	21.50 ± 2.50
EF (%)	34.54 ± 3.67	35.77 ± 3.03	34.50 ± 1.88	37.58 ± 2.94
FS (%)	65.23 ± 4.71	66.62 ± 3.97	64.83 ± 2.25	68.58 ± 3.70
E (cm/s)	0.79 ± 0.10	0.78 ± 0.10	0.86 ± 0.11	0.83 ± 0.15
A (cm/s)	0.52 ± 0.13	0.51 ± 0.13	0.56 ± 0.18	0.58 ± 0.15
AO (cm/s)	1.04 ± 0.13	0.98 ± 0.14	1.00 ± 0.21	1.08 ± 0.12
E/e	7.84 ± 1.58	7.45 ± 1.31	8.30 ± 1.63	8.47 ± 1.50

LVDd, left ventricular end-diastolic diameter; IVS, interventricular septal thickness; LVPW, left ventricular posterior wall thickness; LA, left atrial anterior-posterior diameter; EF, ejection fraction; FS, fraction shortening; E, blood flow velocity through mitral valve during early diastole; A, blood flow velocity through mitral valve during late diastole; AO, blood flow velocity through aortic valve during systole; e, the average of tissue velocities on the septal and lateral sides of mitral annual during early diastole.

Continuous data are presented as the mean ± standard deviation.

### 3.2 Morphological changes in the novel fully biodegradable occluder for VSD closure during the follow-up period by echocardiography

**Figure 2:**
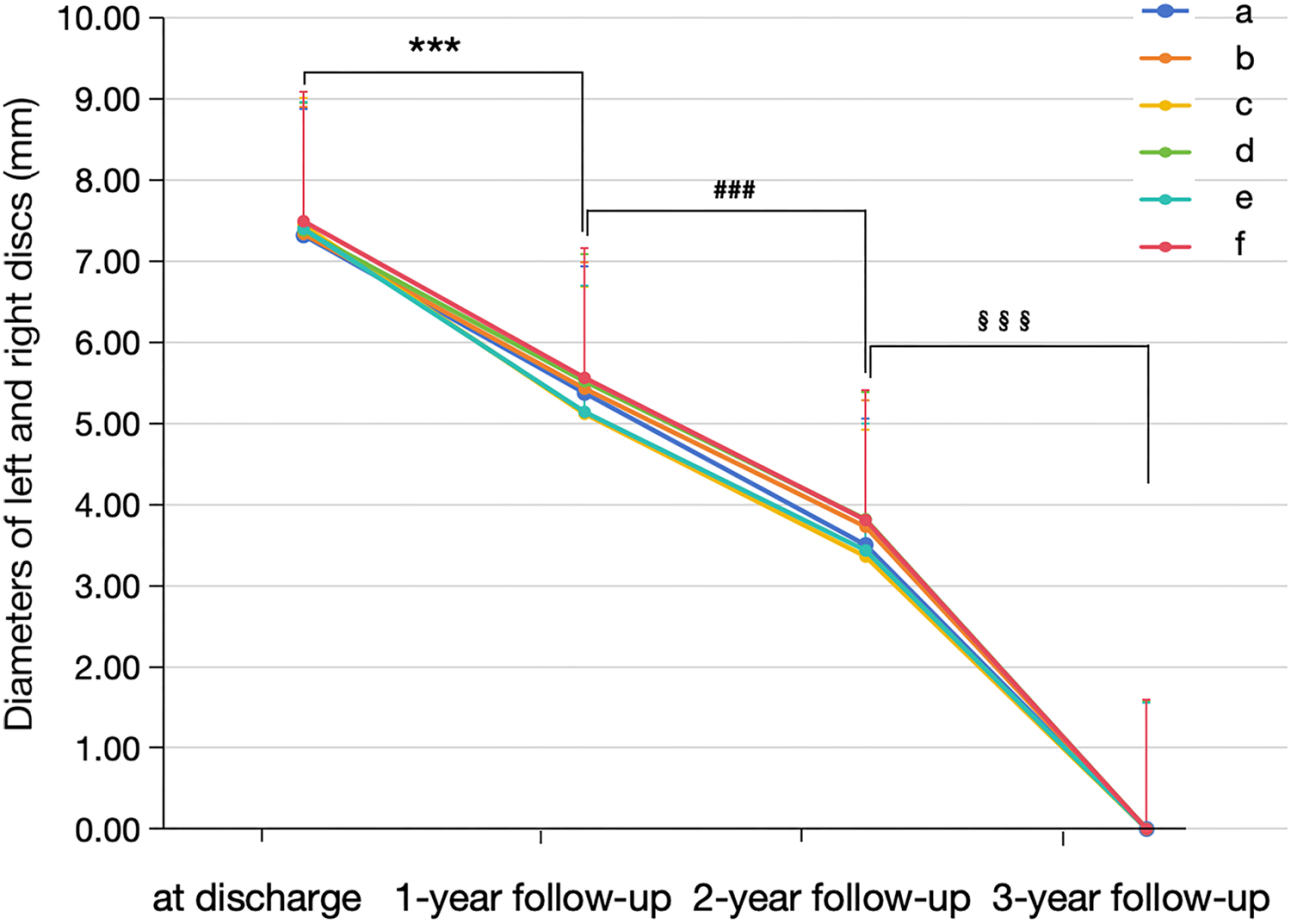
Morphological changes in the novel fully biodegradable occluder for VSD closure during the follow-up. From discharge to the third year, the diameters of the left and right discs of the novel medical devices gradually decreased over time and were eventually invisible under scanning at the end of the third year after implantation. a and b indicate the diameters of the left and right discs in the apical three-chamber view; c and d indicate the diameters of the left and right discs in the apical four-chamber view; e and f indicate the diameters of the left and right discs in the short-axis view. ***p-value < 0.001 vs. at discharge; ^###^*p*-value < 0.001 vs. 1-year follow-up; ^§§§^*p*-value < 0.001 vs. 2-year follow-up.

**Figure 3:**
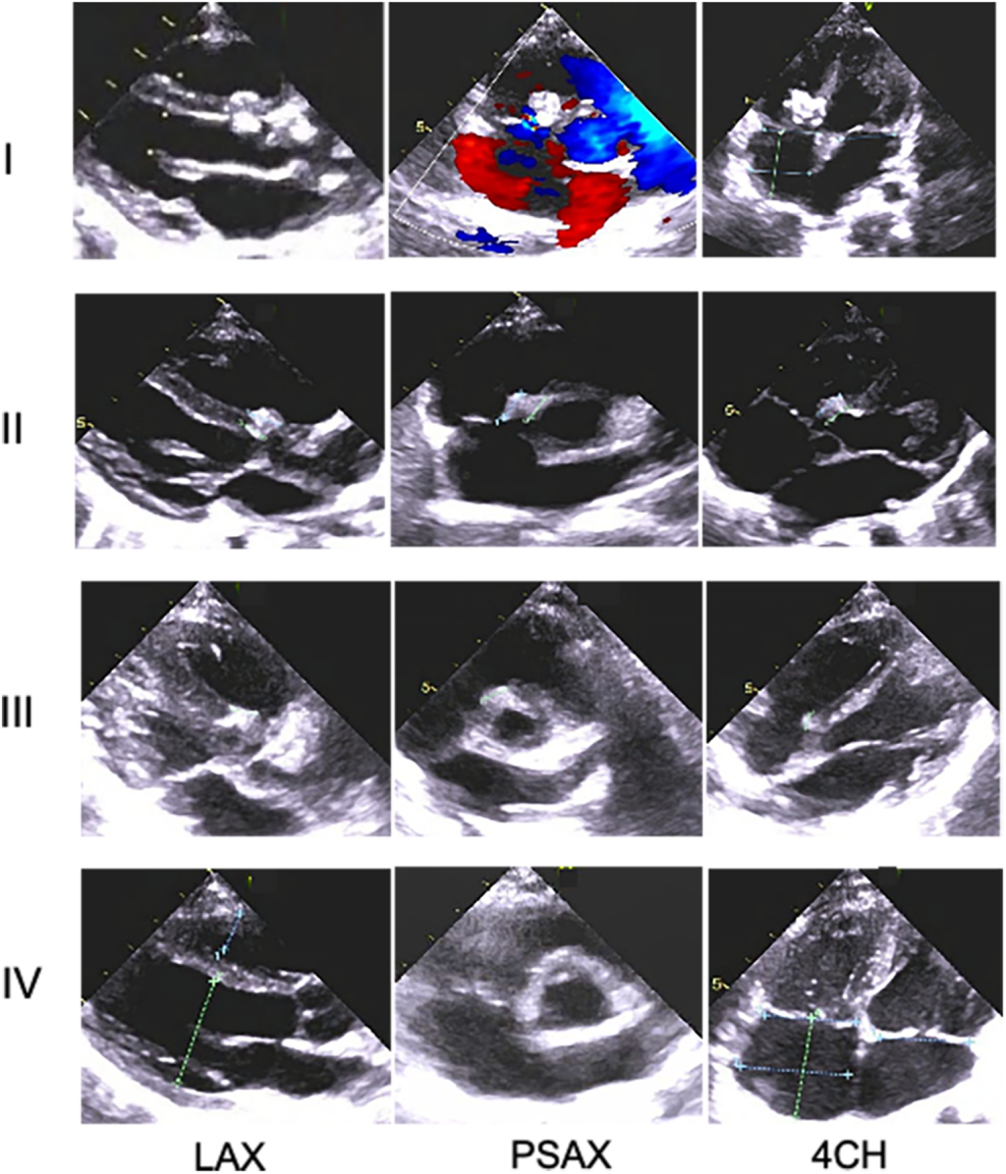
Changes in morphology of the novel device with time in one Pm-VSD patient during the follow-up period using transthoracic echocardiography. At every visit, the occluder remnants tended to gradually decline and could not be identified at the end of follow-up, indicating increasing absorption over time and eventual complete degradation of the novel device. Rows I–IV indicate at discharge and 1-year, 2-year, ,and 3-year follow-up after implantation, respectively; LAX, parasternal long-axis view of the left ventricle; PSAX, parasternal short-axis view of the aorta; 4CH, the apical four chamber.

According to the time intervals between implantation and March 2024, all 30 enrolled cases completed the 1-year follow-up, 24 completed the 2-year follow-up, and 13 completed all three follow-ups. During the follow-up, the size of the left and right discs of the novel medical devices gradually decreased over time and were eventually invisible under echocardiographic scanning in the third year after implantation (Figures 2, 3). Moreover, the length of the left disc decreased slightly faster than that of the right disc, but there was no significant difference between them (Table 4).

**Table 4:** Changes in the lengths of the left and right discs of the novel fully biodegradable occluder for VSD closure during the follow-up period using echocardiography.

Variables (mm)		At discharge	1-year follow-up	2-year follow-up	3-year follow-up
		(*n* = 30)	(*n* = 30)	(*n* = 24)	(*n* = 13)
Apical 3 CH	Left	7.31 ± 0.97	5.37 ± 1.24^a^	3.50 ± 1.41^b^	0.00^c^
	Right	7.33 ± 1.04	5.43 ± 1.27^a^	3.73 ± 1.40^b^	0.00^c^
Apical 4 CH	Left	7.44 ± 1.13	5.12 ± 1.30^a^	3.36 ± 1.47^b^	0.00^c^
	Right	7.38 ± 1.09	5.52 ± 1.28^a^	3.82 ± 1.58^b^	0.00^c^
Short-axis	Left	7.40 ± 1.00	5.14 ± 1.51^a^	3.43 ± 1.73^b^	0.00^c^
	Right	7.49 ± 1.02	5.56 ± 1.46^a^	3.81 ± 1.51^b^	0.00^c^

CH, chamber.

Continuous data are presented as the mean ± standard deviation.

^a^ *p*-value < 0.001 vs. at discharge.

^b^ *p*-value < 0.001 vs. 1-year follow-up.

^c^ *p*-value < 0.001 vs. 2-year follow-up.

### 3.3 Myocardial function characteristics in cases with the novel implantable device at the end of the 3-year follow-up period

At the end of the 3-year follow-up, the myocardial deformation measurements in the ventricular septal basal segments were almost the same as the values of the LV free wall bases among the participants implanted with the novel fully biodegradable devices (*p *> 0.05) (Table 5). In all 13 patients who completed all three follow-ups, the global values of strain along longitudinal (20.85% ± 1.79% vs. 21.04% ± 1.56%), circumferential (18.27% ± 2.06% vs. 18.11% ± 2.39%), and radial (48.05% ± 5.32% vs. 48.21% ± 4.45%) dimensions in 16 segments did not have significant differences when compared to the matched controls (*p* > 0.05).

**Table 5:** Myocardial deformation of cardiac basal segments in the cases after implanting the novel biodegradable device at the end of 3-year follow-up period.

Variables	Ante septum	Anterior	Lateral	Posterior	Inferior	Septum
Basal LS (%)	19.49 ± 1.81	19.54 ± 1.53	19.38 ± 1.61	19.92 ± 1.77	20.08 ± 2.06	19.38 ± 1.55
Basal CS (%)	16.63 ± 1.88	17.02 ± 1.72	17.11 ± 2.09	17.49 ± 1.98	17.38 ± 1.81	16.96 ± 1.76
Basal RS (%)	47.33 ± 4.63	47.35 ± 3.81	47.70 ± 5.33	48.09 ± 5.62	48.12 ± 5.77	46.82 ± 6.04

LS, longitudinal strain; CS, circumferential strain; RS, radial strain.

Myocardial deformation data are presented as the absolute value (mean ± standard deviation).

## 4 Discussion

In the present study, we demonstrated that the fully biodegradable double-disc occluder gradually decreased over time and was eventually absorbed during the 3-year follow-up period. Moreover, the myocardial deformation parameters were not obviously different, not only between the cases implanted with the novel devices and the normal controls, but also between the basal segments of the ventricle septa and of the LV free wall among the patients who completed the Pm-VSD closure using the fully biodegradable occluder. To the best of our knowledge, this is the first long-term follow-up of changes in the morphology of fully bioabsorbable devices over time in children with Pm-VSD and the first report evaluating myocardial deformation characteristics in the cases implanted with the novel occluder at the end of a 3-year follow-up.

Compared to the metal device, the perfect fully biodegradable occluder should meet the following conditions: (1) superior geometric adaptability—the device can fit to different morphologic defects well and clamp the site of defect effectively; (2) excellent time efficiency—the device can provide an accurate balance between the occluder absorption and the tissue regeneration, which acts as a supporting net before the defect is entirely endothelialized, which, after that, is gradually assimilated and eventually degraded completely. Meanwhile, the primary defect is covered and closed using autologous tissue without a residual shunt; and (3) good biocompatibility—it will not cause biotoxic effects to the human body, such as cytotoxicity and genotoxicity (14, 15).

In this 3-year cohort study, the novel fully bioabsorbable device demonstrated its excellent properties for VSD closure. All the Pm-VSDs were clamped successfully without serious complications, including late-onset reactions. During the postoperative follow-up, both the left and right discs of the occluder remained intact and gradually diminished in size over time. At the end of the third year, the devices were absorbed completely without any residual shunt under transthoracic echocardiography scanning. Moreover, it is interesting that the degradation and endothelialization levels of the two sides of the discs were slightly unbalanced. The left disc was absorbed slightly faster than the right, probably because of the more oxygen-rich and higher blood flow in the left ventricle. Those findings were similar to those in a previous report of an animal model and pilot clinical trial including five patients and a 3-month follow-up (11).

We also evaluated the global and segmental myocardial function of the Pm-VSD cases implanted with the bioresorbable polydioxanone device. Previous studies on animal models of VSD treated with the novel bioabsorbable occluder have shown, 36 months postoperatively, that the polydioxanone device was completely degraded accompanied by a reconstructive process of a highly fibrillar micro-structure, which was similar to the architecture of native myocardium without scar formation (9). In the present study, there were almost the same measurements of strain in the basal segments between the septa and the ventricular free wall in the patients at the end of follow-up. The values of strain along the longitudinal, circumferential, and radial dimensions were not remarkably different between the cases with the novel device implantation and the controls. Thus, the novel, fully bioabsorbable occluder for VSD showed perfect biocompatibility and had no adverse effects on the local and global myocardial deformation of the patients using the novel device.

In addition, in the present study, the procedure was undertaken through the minimal right subaxillary incision-right atrial thoracotomy, which did not require extracorporeal circulation and blood transfusion and also avoided radiation damage with a small trauma (11).

The main limitation of this study is the small sample size. In addition, although 3-year follow-up was required until the device was invisible under scanning, a more detailed longitudinal follow-up and analysis is necessary. With the future application of the novel bioresorbable implantation in the clinic, the evaluation of this occluder for VSD closure will be more comprehensive.

## 5 Conclusion

After the 3-year follow-up, the safety, effectiveness, and perfect biocompatibility of the novel fully bioabsorbable polydioxanone occluder for VSD treatment have been confirmed. Echocardiography plays a crucial role in the follow-up of this revolutionary procedure for Pm-VSD closure.

## Data Availability

The original contributions presented in the study are included in the article/Supplementary Material, further inquiries can be directed to the corresponding author.
